# Tungsten and Cobalt: Sheppard et al. Respond

**DOI:** 10.1289/ehp.10614R

**Published:** 2008-05

**Authors:** Paul R. Sheppard, Robert J. Speakman, Gary Ridenour, Mark L. Witten

**Affiliations:** Laboratory of Tree-Ring Research, University of Arizona, Tucson, Arizona, E-mail: sheppard@ltrr.arizona.edu; Museum Conservation Institute, Smithsonian Institution, Suitland, Maryland; General medical practice, Fallon, Nevada; Department of Pediatrics, University of Arizona, Tucson, Arizona

First and foremost, our data from Nevada (Sheppard et al. 2007c) should not be quantitatively compared with that from Oregon. We did not make such a comparison in our article; to reinforce separation of these two studies, we described the results in separate subsections and presented data in separate figures. Our Oregon study was an independent test of dendrochemistry for establishing temporal patterns of environmental tungsten in a town with known emission of airborne tungsten. Tungsten emission in Sweet Home, Oregon, began in November 2000, and tree-ring tungsten in cottonwoods near the emission source increased at that time relative to comparison towns in central Oregon. This test demonstrated that dendrochemistry accurately depicts tungsten availability, at least when using cottonwoods. Therefore, dendrochemistry, especially when using cottonwood trees, can be trusted to accurately depict tungsten availability in Fallon, Nevada, where timing of emission of tungsten particles is not known with certainty.

The comparison of real interest was between Fallon and other towns of west-central Nevada. Tree-ring tungsten in Fallon was not significantly different from that of other towns of west central Nevada during the tree-ring period centered on 1991 [Figure 4 in our article (Sheppard et al. 2007c)], before the onset of the leukemia cluster. During the tree-ring period centered on 1995, corresponding to just before the onset of the leukemia cluster, Fallon tree-ring tungsten began trending upward and was significantly higher than Nevada comparison towns. During the following two time periods, overlapping temporally with the childhood leukemia cluster, Fallon tree-ring tungsten continued trending upward and remained higher than Nevada comparison towns, with significance levels at or near *p* = 0.05 (we provided *p*-values in place of error bars), thus indicating the temporal correspondence between elevated tungsten in Fallon and the childhood leukemia cluster.

The Centers for Disease Control and Prevention (CDC) conclusion that tungsten is not mathematically associated with the leukemia cases of Fallon (CDC 2003) is based on case-comparison testing within Fallon. This does not rule out that an underlying association actually exists but is not detectable by the case-comparison technique. Granted, no relation was reported between leukemia and tungsten exposure, but exposure to tungsten was found to be community-wide, with levels being high both in case children and families and in comparison children and families (CDC 2003). In other words, there was little to no variability in exposure at the community scale (i.e., most everyone in Fallon has been exposed) but high variability in onset of disease (i.e., some people got leukemia but others have not). When variability of an exposure is low relative to individual susceptibility to a disease, genetic studies are needed to identify gene polymorphisms that might make sick children more susceptible to effects of the exposure (Steinberg et al. 2007).

Our environmental research in Fallon has followed an ecologic approach with the philosophy that greater variability in exposure between different towns is more important than the minor variability in exposure within communities (Sheppard et al. 2007a). The entire town of Fallon has been compared environmentally with other towns of west-central Nevada. Multiple environmental indicators have been used, such as outdoor airborne particulates (Sheppard et al. 2006), lichens (Sheppard et al. 2007d), surface dust (Sheppard et al. 2007b), and tree rings (Sheppard et al. 2007c). These indicators incorporate environmental conditions differently from one another, yet they have corroborated one another in showing that airborne tungsten is elevated in Fallon relative to other towns of west-central Nevada or the surrounding desert. Additionally, airborne tungsten particles in Fallon have been identified as anthropogenic in origin, and not natural (Sheppard et al. 2007e).

Even with this preponderance of evidence showing spatial and temporal patterns of airborne tungsten in Fallon, we still have not concluded in any of our reports on Fallon that exposure to tungsten causes leukemia. Quite the opposite: We have acknowledged that environmental data alone cannot lead to such a conclusion and that direct biomedical testing is needed to establish a causal linkage between tungsten and leukemia.

Years ago in an article on disease cluster research, Shimkin (1965) stated that cooperation of industrial management is needed to identify and reduce environmental carcinogens. This comment still rings true today. Kennametal Inc. (Fallon, NV) claims that it supports research in Fallon aimed at understanding the childhood leukemia cluster there (Goodale 2005), but its support is apparently selective. We hereby encourage Kennametal to engage in reasonable dialogue about research in Fallon related to the childhood leukemia cluster.
